# Molecular and biological characterization of pyocyanin from clinical and environmental *Pseudomonas aeruginosa*

**DOI:** 10.1186/s12934-023-02169-0

**Published:** 2023-08-29

**Authors:** Heba Shouman, Heba Shehta Said, Hany I. Kenawy, Ramadan Hassan

**Affiliations:** Department of Microbiology and Immunology, Faculty of Pharmacy, Mansoura University, Mansoura, 35516 Egypt

**Keywords:** Pyocyanin, *Pseudomonas aeruginosa*, Bacterial secondary metabolites, Antitumor activity, Antioxidant activity, Biofilm Inhibition, Biofilm Eradication

## Abstract

**Background:**

Pyocyanin is a secondary metabolite secreted by *P. aeruginosa*. It is a redox-active blue/green phenazine pigment that has various beneficial applications. The present study aims at screening the production of pyocyanin among clinical and environmental *P. aeruginosa* isolates in Dakahlya governorate, Egypt. Thereafter, large-scale production, purification, structure elucidation, and assessment of the biological activity of the highest pyocyanin producers were targeted.

**Results:**

Pyocyanin from the highest clinical (PsC05) and environmental (PsE02) producers were subjected to large-scale production, followed by purification using silica gel column. Pyocyanin was characterized using TLC, UV-Vis, ^1^ H NMR, and FTIR spectroscopy to confirm its structure and purity. Purified pyocyanin showed remarkable antimicrobial efficacy against all tested food-borne pathogens, MDR/XDR clinically isolated bacteria and *C. albicans*. Furthermore, it showed a substantial effect on biofilm inhibition and eradication of pre-formed biofilm against strong biofilm producing bacterial pathogens. However, it had limited antibiofilm activity against *C. albicans*. Pyocyanin from PsC05 had higher antioxidant and radicals scavenging activity than that from PsE02 as determined by FRAP, DPPH, and ABTS assays. Likewise, pyocyanin from PsC05 was more active against tested cancer cell lines, especially human Breast Cancer (MCF-7) and Colorectal Carcinoma (HCT-116), than that from PsE02. More importantly, it showed minimal cytotoxicity to normal cells.

**Conclusions:**

*P. aeruginosa* clinical and environmental isolates produce pyocyanin pigment in varying amounts. Pyocyanin exhibits substantial anti-bacterial, and anti-fungal activity; thus, enhancing its medical applicability. It could be used to inhibit and/or eradicate biofilm from the surfaces of medical devices which is a chief source of nosocomial infections. Its antioxidant along with cytotoxic activity against cancer cell lines, make it a promising contender for use as a substitute for synthetic agents in cancer treatment.

**Supplementary Information:**

The online version contains supplementary material available at 10.1186/s12934-023-02169-0.

## Introduction

*Pseudomonas aeruginosa (P. aeruginosa)* is a gram-negative bacterium that poses a serious threat particularly for individuals with compromised immune systems and in healthcare settings. It has a remarkable set of virulence determinants besides its ability to acquire resistance to numerous antibiotics making it a life-threatening pathogen [1]. Moreover, it is a remarkably adaptable and metabolically versatile microbe that demonstrates broad habitat tolerance, exhibiting ubiquitous distribution in various ecological niches such as soil, water, animals, sewage, hospitals, oil-contaminated areas, and sinks [2, 3].

Microorganisms are fundamental biological entities that are instrumental in addressing environmental, and agricultural problems. They possess a remarkable ability to synthesize novel secondary metabolic products, such as pigments. These pigments not only serve as colorants but also exert biological activities including antimicrobial, anti-biofilm, antitumor, and antioxidant activities. Furthermore, pigments may impart a significant selective advantage by inhibiting the growth of potential microbial competitors [4, 5]. Pigments derived from microbial sources have garnered considerable scientific interest in recent years, owing to their eco-friendly and non-toxic attributes, making them a promising alternative to synthetic pigments used in various applications such as cosmetics, foods, dyes, and pharmaceuticals. Moreover, the utilization of microbial pigments synthesized from waste ingredients provides a sustainable approach for reducing environmental pollution, further highlighting their potential for industrial applications [6].

*P. aeruginosa* generates a substantial diversity of extracellular pigments, where phenazines are the most important [7]. Therefore, it has gained significant attention as one of the most commercially valuable microbial species recognized for its ability to biosynthesize redox-active phenazine derivatives, specifically: pyocyanin, pyomelanin, pyorubrin, and fluorescein [8]. Pyocyanin is a redox-active secondary metabolite with a molecular structure of 5-methyl-1-hydroxyphenazine. It is synthesized during the late stationary phase, imparting a distinct blue hue to the culture [9]. Pyocyanin is considered a virulence factor for *P. aeruginosa* strains. It has the capability to acquire iron from the extracellular environment [10]. It plays a significant role in iron metabolism by actively engaging in reduction mechanisms facilitating the liberation of iron from transferrin [11]. Pyocyanin, the water-soluble pigment, has been shown to exert toxic effects on multiple organs via the production of reactive oxygen species (ROS) [12]. Its toxicity arises from its ability to alter the electron transfer pathways, leading to the generation of excess intracellular oxygen reduction products and subsequent cell death. These findings highlight the complex molecular mechanisms underlying the deleterious effects of pyocyanin on cellular physiology and pathogenesis [12]. Due to its pharmacological impact on both eukaryotic and prokaryotic cells, as well as phytopathogens, pyocyanin holds potential for use as a biocontrol agent [13–15]. Moreover, the anti-biofilm properties of pyocyanin on both established and developing biofilms of different microorganisms have been recorded [16, 17]. In the field of medicine, pyocyanin has shown promising potential as an anticancer agent due to its ability to exert cytotoxic effects on tumor cells. Additionally, it has been demonstrated to possess antioxidant and radical scavenging properties at low concentrations [18]. Therefore, Pyocyanin is a pigment that has a diverse array of prospective applications in areas including medicine, food, pharmaceuticals, biocontrol, nanotechnology, textiles, and physiochemistry. This versatility and multi-functionality make pyocyanin a highly promising substance in various fields [19].

Given the manifold beneficial applications of pyocyanin, the aim of this study is to assess the antimicrobial, anti-biofilm, anticancer, and antioxidant activity of pyocyanin produced by *P. aeruginosa* clinical and environmental isolates in Dakahlya governorate, Egypt. The outcomes of this research could aid in identifying potential sources and applications of pyocyanin in various domains.

## Materials and methods

### Clinical and environmental *P. aeruginosa* isolates

One hundred and twenty-five clinical *P. aeruginosa* isolates were collected from Mansoura University Hospitals in Mansoura, Dakahlya governorate, Egypt. Specimens were collected between October 2016 and May 2017 from different clinical sources including wound, urine, blood, and diabetic foot (Table S1). In addition to the clinical isolates, twenty-five environmental isolates of *P. aeruginosa* were collected from cultivated agricultural lands in Dakahlya governorate, Egypt. The environmental samples were obtained from a depth of 35 cm and subjected to rigorous aseptic handling procedures. Adequate microbiological laboratory techniques were employed to identify the isolates [20] including: streaking on cetrimide agar plates, examination of colonies under a microscope after Gram staining and biochemical identification. Subsequently, *P. aeruginosa* isolates were stored in Mueller Hinton Broth (MHB) containing 20% (v/v) glycerol at -80 °C.

### Screening of pyocyanin production among *P. aeruginosa* isolates

All tested clinical and environmental *P. aeruginosa* isolates were cultured overnight in nutrient broth. Cultures’ optical density was adjusted to 0.2 at 600 nm. Fifty microliters of adjusted cultures were used to inoculate king’s A broth (5ml) followed by incubation for 72 h at 37°C with shaking (200 rpm). Green cultures were centrifuged at 8000 rpm via cooling centrifuge, chloroform (3 ml) was mixed with cell free filtrate and then centrifuged at 8000 rpm. The blue-colored chloroformic layer was transferred to fresh tube containing 0.2 N HCl (1 ml), mixed till development of pink color in the aqueous layer, and then centrifuged again. To quantitatively assess pyocyanin production, absorbance of the acidic pink layer was measured at 520 nm and the concentration was calculated according to the equation: Concentration of pyocyanin (ug/ml) = OD_**520**_ × 17.072 [21]. Isolates from clinical and environmental sources that showed the highest production of pyocyanin were selected for further studies.

### Production, extraction and purification of pyocyanin from selected *P. aeruginosa* isolates

For large-scale production of pure pigment from selected isolates, they were cultured in 500ml conical flask containing 100ml of King’s A broth, incubated for 72 h at 37 ℃ with continuous shaking at 200 rpm. Extraction of acidic pink aqueous layer was performed as previously described, followed by dropwise addition of 1 N NaOH to pink solution until the color changed to blue. Pyocyanin was extracted with chloroform from the blue alkaline solution, and the procedure was repeated three times. Chloroform was evaporated and needle-shaped pyocyanin crystals were collected and kept in sterile opaque container at 4 ℃ [22]. Crude pyocyanin needles were dissolved in chloroform and applied onto silica gel column (30 cm length x 3 cm diameter) equilibrated with 1% methanol in chloroform. Elution of pure pigment was performed using 15% methanol in chloroform. All fractions with blue color were gathered, then dried at 37 ℃ using rotary evaporator.

### Structural elucidation and Purity Assessment of purified pyocyanin

#### Thin Layer Chromatography (TLC)

Using a capillary tube, purified pyocyanin was applied onto TLC plate (Silica gel 60 F254 Aluminum Sheet). The plate was positioned in a glass chamber with mobile phase consisting of methanol: chloroform (1:1) and allowed to run till the solvent reached the end of the plate. After 5 min of drying, the retention factor (R_f_) was determined.

#### UV-Vis spectrophotometry

Purified pyocyanin was dissolved in methanol and 0.1 N HCL, then characterized by UV-Vis Spectrophotometer (Shimadzu UV-1601PC, Japan) over the range 200–700 nm, where absorption maxima were determined.

#### ^1^H NMR spectrometry

Pure pyocyanin was subjected to ^1^H NMR spectroscopy using 400 MHz NMR spectrometer (NMR Center, Faculty of Pharmacy, Mansoura University) using CdCl_3_ HPLC grade as solvent at 25 ℃.

#### Fourier Transform Infra-Red Spectroscopy (FTIR)

Functional groups were identified via FTIR. Translucent sample disks were prepared by encapsulating 200 mg of purified pyocyanin in KBr (Sigma-Aldrich) and homogenizing the mixture. The spectra were measured in the IR region using a Nicolet FTIR 6700 instrument (Thermo Fisher Scientific) with a single beam splitter and a DTGS-KBr detector. The spectrometer was set up to operate in a range of 4000 to 200 cm^− 1^.

### Antimicrobial activity of pyocyanin

#### Test microorganisms

Antimicrobial activity of purified pyocyanin was assessed on indicator strains including:

- food borne pathogens (including *K. pneumoniae, E. coli, K. oxytoca, E. cloacae* and *E. aerogenes*) isolated from dairy products and processed meat.
- human pathogenic MDR/XDR Gram-Negative bacteria (including *K*. *pneumoniae, E. coli*, *P. mirabilis*, and *A. baumannii)* and MDR Gram-Positive bacteria (including *S. pyogens*, *S. agalactiae*, *S. aureus*, and Methicillin-resistant *Staphylococcus aureus* (MRSA).
- human pathogenic *Candida albicans* were also obtained from different sources.

All indicator strains underwent identification steps, which involved Gram staining and assessment of their biochemical activity [20].

#### MIC of pyocyanin

Pyocyanin’s antibacterial activity was assessed against both food-borne and human pathogens (indicator strains) by microbroth dilution method in microtiter plates as previously described [23]. In brief, one colony of each indicator strain was inoculated into nutrient broth and incubated at 37˚C till the turbidity reached approximately 0.5 McFarland standard (1.5 × 10^8^ CFU/ml). Cultures were further diluted to reach final inoculum of 1.5 × 10^5^ CFU/well. Serial dilutions of purified pyocyanin (20–350 µg/ml) were prepared in a microtiter plate and inoculated with indicator strains followed by incubation at 37 ℃ for 24 h. Plates were inspected for growth of the inoculum. MIC was estimated as the lowest concentration of pyocyanin that inhibited microbial growth under specified conditions. The assay was performed in triplicate. Negative and positive controls were included in each experiment. Pyocyanin antifungal activity was assessed using the same method using YPD broth for cultivation of *C. albicans* isolates.

### Anti-biofilm activity of pyocyanin by tissue culture plate method

Efficacy of purified pyocyanin to inhibit biofilm formation and to eradicate pre-formed biofilm by strong producers (including food-borne and human pathogens) was assessed in vitro using tissue culture plate assay method as previously described with minor modifications [24].

#### Biofilm Inhibition Assay

Single colony of indicator strains (pre-grown on tryptic soy agar plates) was used to inoculate 5ml of tryptic soy broth supplemented with 1% anhydrous glucose (TSBG) then incubated overnight at 37 ℃. Cultures were adjusted to reach 0.5 McFarland standard (1.5 × 10^8^ CFU ml^− 1^). For untreated isolates, 100 µl of adjusted culture was dispensed into wells of sterile 96-well microtiter plates containing 100 µl (TSBG). For treated isolates, 100 µl of adjusted bacterial culture were dispensed into wells containing 100 µl of pyocyanin at final sub-MIC concentration (1/2, 1/4, 1/8, and 1/16 MIC). Plates were incubated at 37 ℃ for 24 h. Wells were aspirated and rinsed with sterile saline solution three times to remove non-adherent microorganisms. The attached bacteria were treated with 200 µl of 99% methanol for 15 min for fixation, then wells were decanted and left to dry. Biofilm was stained for 15 min with 200 µl of crystal violet stain (1% w/v), followed by rinsing the plate with water. After drying, 200 µl of glacial acetic acid solution 33% was added to resolubilize the bound dye. Optical Density (OD) at 570 nm was measured using ELISA plate reader (BioTek Instruments Inc., Winooski, VT). Negative control wells consisted of TSBG only. The assay was performed in triplicate. Percentage inhibition of biofilm formation was calculated according to the following equation.

Percentage Inhibition = (OD _untreated_ – OD _treated_)/ OD _untreated_ x 100.

#### Biofilm Eradication Assay

Eradication of pre-formed biofilm was assessed using the same method, permitting the microorganism to form biofilm, after 24 h incubation at 37 ℃, the wells were decanted, rinsed with physiological solution three times under aseptic conditions, and left to dry. Pyocyanin was dispensed into wells at final sub-MIC concentration (1/2, 1/4, 1/18, and 1/16 MIC). The plates were incubated overnight at 37 ℃. Biofilm was stained, and the percentage eradication of pre-formed biofilm was calculated as described previously.

### Evaluation of Biological activity of pyocyanin

#### Antioxidant activity evaluation

Three distinct assays were used to assess free radical scavenging activity of purified pyocyanin including: (FRAP), (DPPH) and (ABTS) assays.

##### FRAP Assay

The assay was conducted according to protocol by [25], with minor modifications. To prepare FRAP reagent, acetate buffer (pH 3.6), TPTZ solution (10 mM), and FeCl_3_ solution (20 mM) were mixed in a 10:1:1 ratio. Dilutions of Trolox solution with concentration of 3 mM in methanol were prepared. Purified pyocyanin (1 mg/ml) was dissolved in DMSO: methanol (10:90). Dissolved pyocyanin (10 µl) was mixed with 190 µl of TPTZ reagent in a 96-well plate, the mixture was kept in dark for 30 min before the intensity of its blue color was measured spectrophotometrically at 593 nm. The experiment was performed in triplicate per treatment and Mean ± SD was recorded.

##### DPPH Radical Scavenging Assay

DPPH assay was performed according to [26]. In a 96-well plate, serial concentrations of purified pyocyanin (dissolved in DMSO and diluted in methanol) were mixed with freshly prepared DPPH reagent (1% in methanol). The mixture was kept for 30 min in dark at room temperature, after which the decrease in color intensity of DPPH was measured spectrophotometrically at 540 nm. Ascorbic acid was used as positive control. The experiment was performed in triplicate for each pyocyanin concentration. Mean ± SD was recorded, and percentage inhibition was calculated according to the equation: Percentage inhibition = (A_control_-A_test_)/A_control_ x 100.

##### ABTS Radical Scavenging Assay

Assay was performed according to [27] with minor modifications. Fresh solution of ABTS radical cation was prepared by mixing equal volumes of 7 mM ABTS stock solution with 3.5 mM potassium persulfate and incubated in the dark for 16–24 h, resulting in a blue green ABTS solution. Small amount of ABTS solution was mixed with methanol to reach an absorbance reading 0.7-1.0 at 734 nm. Activity of the purified pyocyanin was assessed by adding 1.5 ml ABTS solution to 10 µl of different concentrations (100–1000 µg/ml) of pyocyanin. After incubation for 15 min, the change in absorbance at 734 nm was measured. Ascorbic acid was used as positive control. The reduction in absorbance was used to calculate the percentage of inhibition according to the equation: Percentage inhibition = (A_control_-A_test_)/A_control_ x 100.

#### In vitro cytotoxicity evaluation

##### MTT Assay

3-(4,5-Dimethylthiazol-2- yl)-2,5-Diphenyltetrazolium Bromide Assay was used to assess cell viability of different cell lines including: Hepatocellular carcinoma (HEPG-2), Epithelioid Carcinoma (Hela), Colorectal carcinoma Colon cancer (HCT-116), human breast cancer (MCF-7) and prostate cancer (PC-3), Wi-38, and WISH after treatment with pyocyanin at different concentrations to show its cytotoxic effect.

In MTT colorimetric assay, the mitochondrial succinate dehydrogenase is used by living cells to convert a yellow tetrazolium salt to a purple formazan product. The cell lines were incubated in RPMI-1640 broth supplemented with 10% fetal bovine serum, 100 µg/ml streptomycin, and 100 µg/ml penicillin in 5% CO_2_ incubator at 37 °C. Cell lines were inoculated in a 96-well plate (1 × 10^4^ cells/well), then incubated at 37 °C for 48 h in 5% CO_2_ incubator. After incubation, cells were treated with different concentrations of pyocyanin and incubated for an additional 24 h. Following that 5 mg/ml MTT solution was added to each well and the plates were further incubated for 4 h. After incubation, the formed purple formazan crystals were dissolved in DMSO (100 µl). Absorbance was measured at 570 nm using ELISA plate reader. The relative cell viability was calculated as: (A _treated samples_/A _untreated sample_) x100.

## Results and discussion

### Screening of pyocyanin production among *P. aeruginosa* isolates

Clinical and environmental isolates of *P. aeruginosa* were examined for their ability to produce pyocyanin pigment (Table S1). Out of the 125 tested *P. aeruginosa* clinical isolates, 57 isolates (45.6%) were pyocyanin producers while 68 isolates (54.4%) did not produce pyocyanin (Fig. S1A). Forty-six isolates (36.8%) produced low-level pyocyanin pigment (< 5 µg/ml), nine isolates (7.2%) produced moderate levels (5–10 µg/ml), and only two isolates (1.6%) produced a high amount of pyocyanin (> 10 µg/ml).

Among the tested 25 environmental isolates, 14 environmental isolates were unable to produce the pyocyanin pigments, one isolate produced low level, nine isolates produced moderate levels, and only one isolate produced high level of pyocyanin pigment (> 10 µg/ml) (Fig. S1B). Statistical analysis has indicated that high/moderate levels of pyocyanin production were more common among environmental isolates than clinical ones (chi-square statistic is 22.1629 and p-value is < 0.001). A previous investigation indicated that pyocyanin production among *P. aeruginosa* isolates obtained from rice-growing soil and urinary tract infections were 9.3 µg/ml and 5.9 µg/ml, respectively [7]. The heterogeneity in the synthesis of pyocyanin among *aeruginosa* strains may be attributed to acyl-homoserine lactone (AHL) autoinducers, which are implicated in regulating the transcription and modification of pyocyanin. Pyocyanin production increases because of increase in AHL. Environmental conditions such as pH, oxygen tension, temperature, and oxidative stress, also significantly impact pyocyanin production [28].

### Purification and structure elucidation of pyocyanin

The highest pyocyanin producing clinical isolate (PsC05) and environmental isolate (PsE02) were selected for large-scale production, extraction, and purification of pyocyanin pigment. Thin Layer Chromatography (TLC) analysis of purified pyocyanin showed one single spot with R_f_ of 0.8. The established R_f_ of standard pyocyanin pigment is normally between 0.70 and 0.81 [22].

**Fig. 1:**
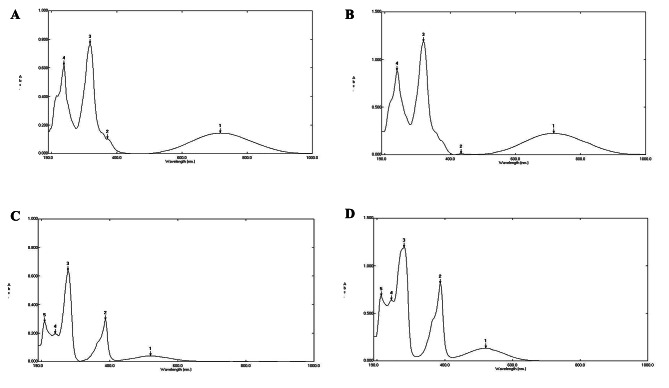
UV-Vis absorption spectrum of purified pyocyanin from PsC05 and PsE02 isolates. **A**: pyocyanin from PsC05 dissolved in methanol. **B**: pyocyanin from PsE02 dissolved in methanol. **C**: pyocyanin from PsC05 dissolved in 0.1 N HCl. **D**: pyocyanin from PsE02 dissolved in 0.1 N HCl.

Purified pyocyanin pigment absorbance spectrum was observed using UV-Vis spectrophotometer (190–900 nm). Pyocyanin from PsC05 and PsE02 dissolved in methanol, had displayed absorption peaks at about 237.5, 318, 434 and 700 nm (Fig. 1A and B); while pyocyanin dissolved in 0.1 N HCl displayed peak at about 212, 242.5, 280, 387, and 521 nm (Fig. 1C and D). These results were in accordance with previous reports [29, 30]. The UV-Vis spectrum of pyocyanin showed characteristic peaks at 201, 238, 318.5, 710.5 and 886.5 nm when dissolved in methanol. While it showed distinctive peaks at 204, 242.5, 277, 387.5 and 512.5 nm when dissolved in 0.2 M HCl [29].

**Fig. 2:**
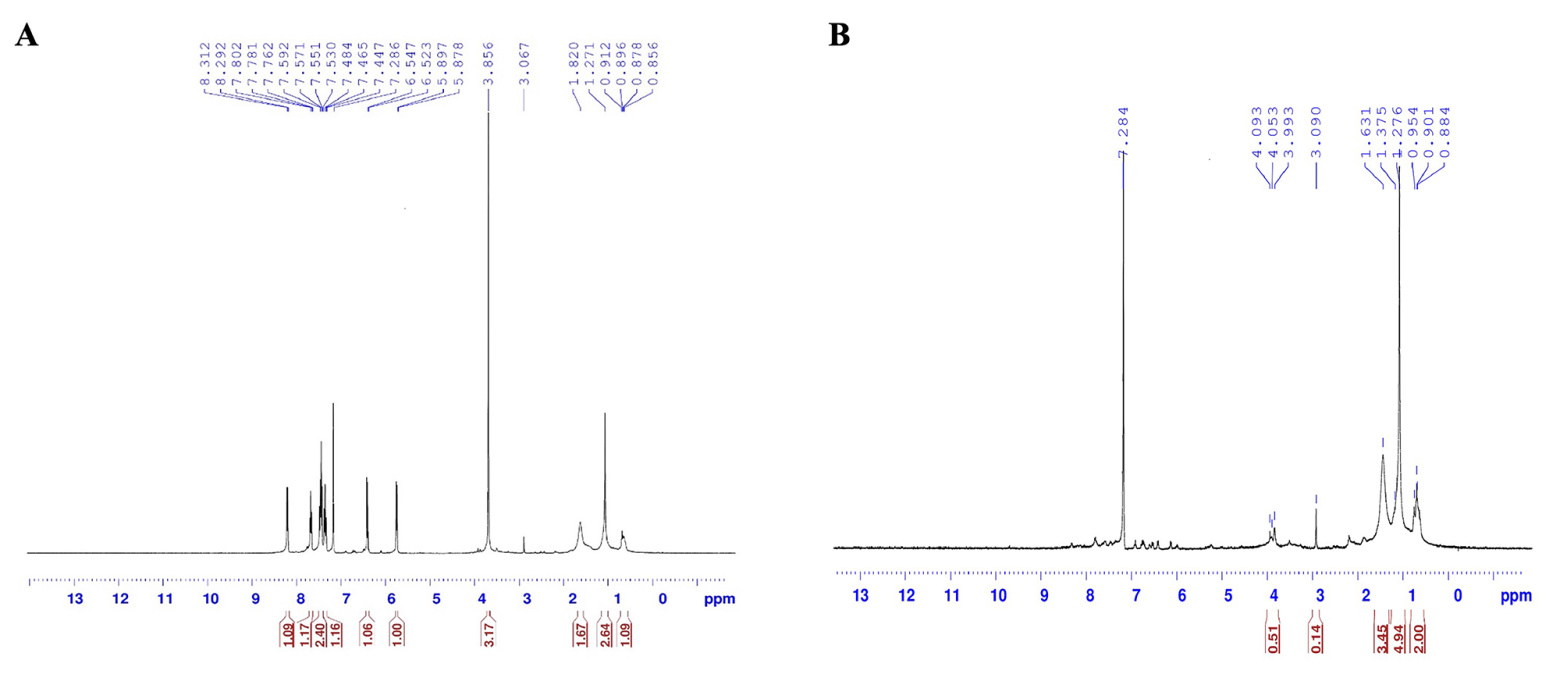
^1^H-NMR spectrum of purified pyocyanin from PsC05 **(A)** and PsE02 **(B)** isolates

Structural elucidation of pyocyanin was assessed using nuclear magnetic resonance (NMR) spectroscopy (Fig. 2A and B). The resulting NMR stretches displayed two distinctive peaks, one of which was a singlet resonating at 3.856 ppm, while persistent protons were detected within the aromatic region, exhibiting multiplets spanning from 7.286 to 7.802 ppm in resonance. These results were in agreement with previous findings indicating purity of the tested pyocyanin [7, 14].

**Fig. 3:**
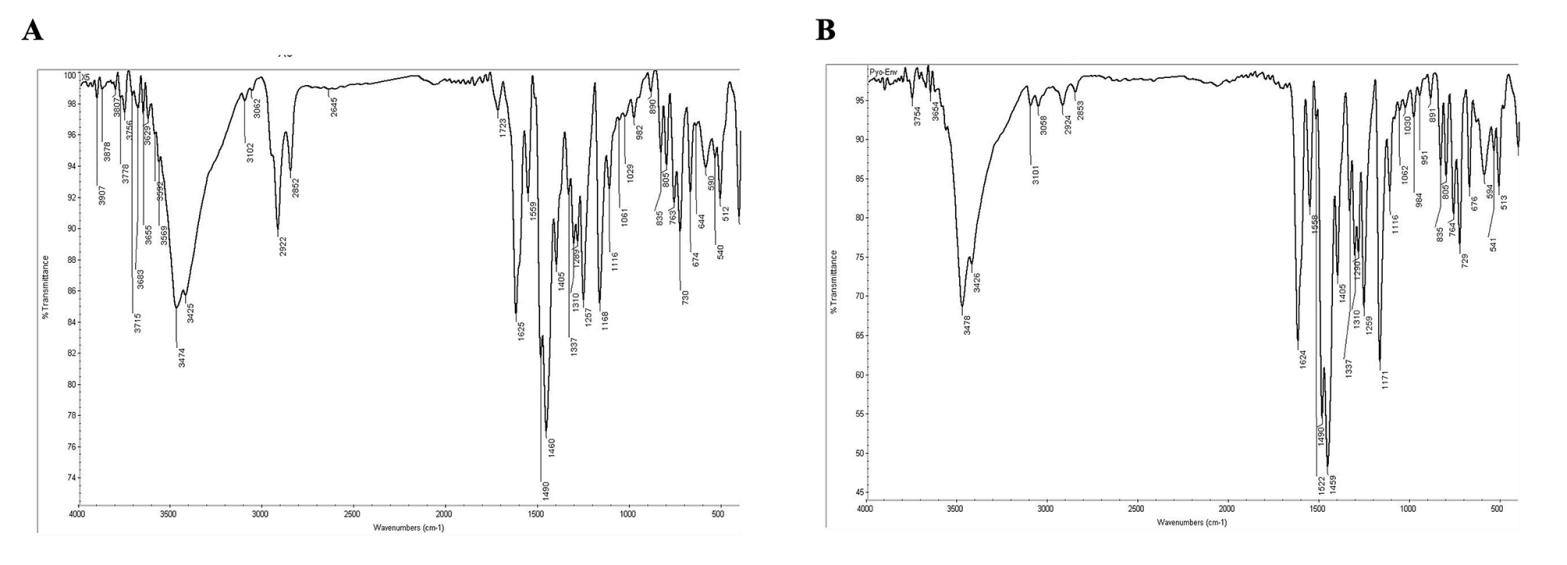
*FTIR* spectral annotation of functional groups of purified pyocyanin from PsC05 **(A)** and PsE02 **(B)** isolates

FTIR analysis of standard pyocyanin showed an alkenyl C = C bond vibration at 1604 cm^− 1^, a C = O-H vibration at 1405 cm^− 1^ suggesting the presence of an aldehyde, and a C-C-C bond vibration at 1169 cm^− 1^ [4]. The analyzed samples displayed similar molecular functional groups at 1625 cm^− 1^, 1405 cm^− 1^, and 1168 cm^− 1^, consistent with the established standard (Fig. 3A and B).

### Anti-microbial activities of pyocyanin

**Fig. 4:**
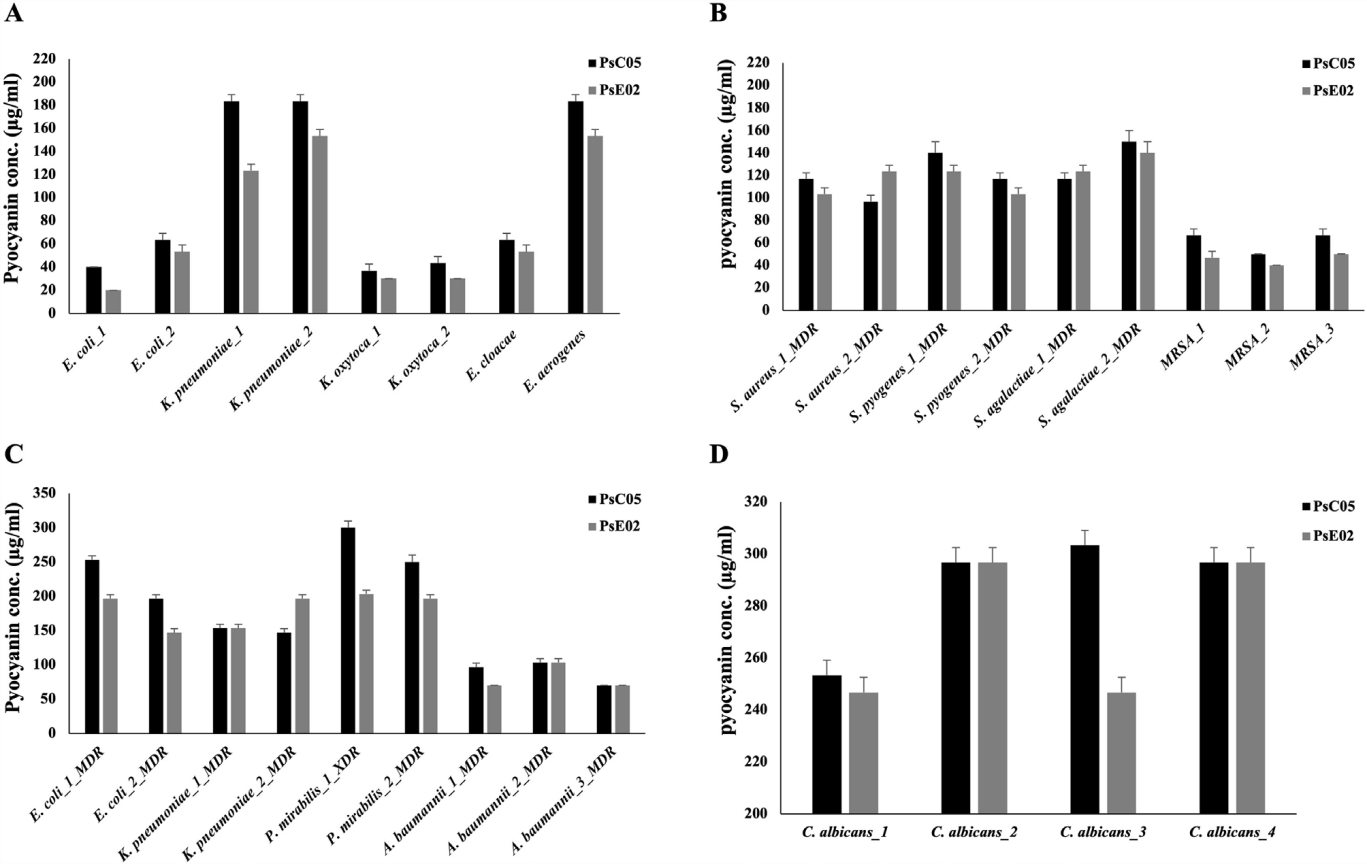
Antimicrobial activity of purified pyocyanin from PsC05 and PsE02 isolates against food-borne pathogens **(A)**, human pathogenic Gram-Postive **(B)** and Gram-Negative **(C)** bacteria, and human pathogenic *C. albicans***(D)**

Antimicrobial activity of purified pyocyanin derived from PsC05 and PsE02 isolates were tested (Fig. 4 and Table S2). Purified pyocyanin showed substantial antimicrobial activity against Gram-Positive and Gram-Negative pathogenic, and food-borne pathogens. Among food-borne pathogens, pyocyanin showed strong activity against *E. coli*, *K. oxytoca*, and *E. cloacae*, with MIC values ranging from 20 to 60 µg/ml. Alternatively, low activity was recorded against *K. pneumoniae* and *E. aerogenes*, with MIC values ranging from 120 to 180 µg/ml (Fig. 4A). A previous study reported MIC value of 183.4 µg/ml for pyocyanin against *K*. *pneumoniae* [8]. Another study indicated that pyocyanin displayed antimicrobial activity towards standard food-borne pathogens at 50 ng/µl [16].

Regarding pathogenic microorganisms, Pyocyanin exhibited superior activity against MRSA, with MIC value of 40–70 µg/ml. Meanwhile, lower antimicrobial activity was detected against MDR *S. aureus*, *S. pyogens*, and *S. agalactiae*, with MIC values 100–150 µg/ml (Fig. 4B). Among Gram-negative pathogens, the best activity was recorded against *A. baumannii* with MIC values 70–100 µg/ml. Higher MIC values were recorded against *E. coli*, *K. pneumoniae*, and *P. mirabilis* ranging from 150 to 300 µg/ml (Fig. 4C). A previous study reported no visible growth of *S. aureus* and *Klebsiella sp*. at concentration 12 and 8 µg/ml of pyocyanin, respectively [31]. While in another study, pyocyanin showed intermediate activity against Gram-Negative bacteria, including *P. mirabilis* and *S. typhi*. Conversely, *K. pneumoniae* was completely resistant towards pyocyanin, while *E*. *coli* displayed high sensitivity towards it [32].

Based on our results, pyocyanin was more effective against Gram-Positive bacteria than Gram-Negative ones. This observation aligns with previous reports [7]. It is noted that the sensitivity of bacteria to pyocyanin may vary based on the amount of lipids present in the cell walls of Gram-Positive and Gram-Negative bacteria [33]. This highlights the importance of considering the structural differences between these bacterial groups in evaluating the efficacy of antimicrobial agents.

Pyocyanin was evaluated for its antifungal potential against human pathogenic *C. albicans*. MIC of pyocyanin was in the range of 250–300 µg/ml (Fig. 4D). The suppression of growth of *C. albicans* and other harmful fungi including other *Candida* species, and *A. fumigatus*, was noted by numerous clinical isolates of *Pseudomonas*. *P*. *aeruginosa* secretes antifungal agents, including the redox active molecule pyocyanin, which has been identified as a potent antifungal compound with a lower minimum inhibitory concentration compared to amphotericin B or fluconazole against *C. albicans*. The recent discovery of the “red death” phenomenon in *C. albicans* caused by *Pseudomonas* has been linked to the antifungal properties of 5-methyl-phenazine-1-carboxylic acid, a pyocyanin precursor [34]. A previous report stated that MIC of pyocyanin against *C. albicans* and *A. fumigatus* was approximately 64 µg/ml [35]. Another investigation revealed that the growth of *Magnaporthe grisea* was completely suppressed at 150 ppm of pyocyanin [4].

### Anti-biofilm activity of pyocyanin

Biofilm is a complex, organized structure composed of proteins, nucleic acids, and exopolysaccharides. It has a pivotal contribution to the emergence and proliferation of antimicrobial resistance [36]. Conventional antibacterial agents face a significant challenge in treating infections caused by biofilm forming bacteria as bacteria within the biofilm demonstrate increased resistance [17]. The establishment of compounds to impede the formation of bacterial biofilms or eradicate established biofilms is of significant importance in the effective management of such infections [37]. Phenazines and their derivatives are thought to have antibiofilm activity. This surging scientific curiosity arises from the revelation that pyocyanin promotes the superiority of biofilm infections induced by *P. aeruginosa* over biofilm infections by *S. aureus* in lung tissues of individuals diagnosed with cystic fibrosis [38]. The effect of pyocyanin sub-MIC on inhibition and eradication of biofilm formed by strong biofilm-producing foodborne and human pathogens was evaluated.

### Inhibition of biofilm formation

Pyocyanin from PsC05 and PsE02 exhibited substantial activity against strong biofilm producing foodborne and human pathogens. The inhibitory activity of ½ MIC of pyocyanin ranged from 61 to 83% inhibition of biofilm formation compared to untreated controls (Table S3). The activity decreased as the concentration of pyocyanin decreased from ½ to 1/16 MIC of each tested organism, where 1/8 MIC showed very limited activity and 1/16 MIC almost failed to inhibit biofilm formation compared to controls. On the other hand, ½ MIC showed moderate inhibitory activity (34 to 55%) against biofilm of tested *C. albicans*. While 1/8 and 1/16 MIC almost failed to inhibit biofilm formation (Table S3).

### Eradication of pre-formed biofilm

Like biofilm inhibitory activity of pyocyanin from PsC05 and PsE02, they exhibited considerable activity in eradication and destroying of pre-formed biofilm of food-borne and human pathogens. The inhibitory activity of ½ MIC of pyocyanin from both isolates was up to 73% compared to untreated controls (Table S4). The activity decreased as the concentration of pyocyanin decreased, where 1/8 and 1/16 MIC showed very limited or almost failed to inhibit biofilm formation. On the contrary, ½ MIC showed very limited inhibitory activity (25 to 34%) against pre-formed *C. albicans* biofilms. Whereas 1/8 and 1/16 MIC completely failed to inhibit biofilm formation (Table S4).

In a prior research, pyocyanin pigment extracted from aquatic environment exhibited significant inhibitory effect on biofilm formation by *B. cereus, S. aureus* and *K. pneumoniae* at 50 µg/ml [16]. Another study demonstrated that 10 ng/µl of pyocyanin failed in inhibiting biofilm formation by *B. amyloliquefaciens CD16* and *B. velezensis 5.* Nevertheless, 50 and 100 ng/µl effectively reduced biofilm formation by 80% for both bacterial strains. In addition, 10 ng/µl was effective in suppressing of 50% biofilm formed by *B. subtilis subsp*. *subtilis MBGLi97*. Higher concentrations (50 and 100 ng/µl) showed 80% reduction in biofilm formation [17].

### Evaluation of Biological activity of pyocanin

#### Antioxidant activity

Reactive oxygen species (ROS) possess the capability of damaging cellular organelles/structures, by means of oxidative injury of lipids, proteins, and DNA. This form of injury is a significant factor in the etiology of a lot of pathological conditions including: neurodegenerative diseases, metabolic disorders, cardiovascular diseases, and malignancies. Recently, scientific research has focused on discovering novel antioxidants that possess high potency and safety profiles, as a strategy to counteract oxidative damage and decrease the incidence of these debilitating illnesses [39]. In this study, three distinct antioxidant tests were used to determine the antioxidant activity of purified pyocyanin (Table 1).

**Table 1:** Antioxidant activity of purified pyocyanin from PsC05 and PsE02 isolates

pyocyanin	FRAP TEAC (µM/mg)	ABTS IC_50_ (µg/ml)	DPPH IC_50_ (µg/ml)
Pyocyanin from PsC05	171.12 ± 5.67	52.73 ± 0.25	45.41 ± 0.2
Pyocyanin from PsE02	109.75 ± 9.7	64.53 ± 0.32	56.18 ± 0.29
Ascorbic acid*	------	25.79 ± 0.16	16.81 ± 0.10

*Ascorbic acid acts as standard antioxidant in (ABTS) and (DPPH) assays

Data expressed as Mean ± SD.

##### FRAP Assay

The ferric ion reduction potential of pyocyanin was evaluated by means of the Trolox equivalent antioxidant capacity technique, presented as µM TEAC/mg of pyocyanin. Pyocyanin from PsC05 displayed high activity with a value of 171.12 ± 5.67, while pyocyanin from PsE02 showed lower activity of 109.75 ± 9.7.

##### ABTS Assay

Pyocyanin from PsC05 (IC_50_ 52.73 ± 0.25) had higher antioxidant activity than pyocyanin from PsE02 (IC_50_ 64.53 ± 0.32). While IC_50_ value of ascorbic acid was 25.79 ± 0.16.

##### DPPH Assay

Pyocyanin from PsC05 exhibited higher DPPH radicals scavenging activity (IC_50_ 45.41 ± 0.2) than pyocyanin from PsE02 (IC_50_ 56.18 ± 0.29). IC_50_ of ascorbic acid was 16.81 ± 0.10.

Sengupta and coauthors recorded that pyocyanin has remarkable antioxidant and free radical scavenging activity upon testing by ABTS, DPPH and FRAP assays [40]. Another study indicated that pyocyanin exhibited substantial antioxidant activity in neutralizing free radicals, even at extremely low concentrations, which suggests a promising safety of using pyocyanin as antioxidant [41].

#### In vitro cytotoxicity evaluation

Microbes produce natural products, including secondary metabolites and antibiotics, in response to environmental challenges, which can have therapeutic benefits for humans. These products could have antitumor activity and be implemented in management of various malignancies [42]. Toxicity testing is a pivotal measure in the evaluation of any new substance. Therefore, crude plant extracts, natural products, or microbial secondary metabolites should not be assumed safe for use without prior cellular toxicity testing [43].

MTT assay was employed to evaluate the cytotoxic activity of purified pyocyanin from PsC05 and PsE02 in vitro against five cancer cell lines (HePG-2, MCF-7, HCT-116, Hela, and PC-3) and two normal cells (WI-38 and WISH). Doxorubicin served as a positive control. IC_50_ values were used to express the concentration at which 50% of the cell monolayer was lost (Table 2, Fig. S2).

**Table 2:** Evaluation of cytotoxicity of purified pyocyanin from PsC05 and PsE02 isolates

	In vitro Cytotoxicity (IC_50_ µg/ml) *						
	HePG2	HCT-116	Hela	MCF-7	PC3	WI38	WISH
DOX**	4.50 ± 0.2	5.23 ± 0.3	5.57 ± 0.4	4.17 ± 0.2	8.87 ± 0.6	6.72 ± 0.5	3.34 ± 0.2
**PsE02**	61.71 ± 3.4	53.06 ± 3.1	74.13 ± 3.9	37.29 ± 2.2	63.21 ± 3.3	85.47 ± 4.1	> 100
**PsC05**	46.45 ± 2.7	32.34 ± 2.1	39.27 ± 2.3	28.85 ± 1.9	41.31 ± 2.3	92.23 ± 4.5	> 100

* IC_50_ (µg/ml): 1–10 (very strong), 11–20 (strong), 21–50 (moderate), 51–100 (weak), and above 100 (non-cytotoxic)

** DOX: Doxorubicin

IC_50_ for pyocyanin from PsC05 was 46.45 ± 2.7, 32.34 ± 2.1, 39.27 ± 2.3, 28.85 ± 1.9, and 41.31 ± 2.3, while IC50 of pyocyanin from PsE02 was 61.71 ± 3.4, 53.06 ± 3.1, 74.13 ± 3.9, 37.29 ± 2.2, and 63.21 ± 3.3 towards HePG2, HCT-116, Hela, MCF-7 and PC-3, respectively. IC_50_ value of pyocyanin from PsC05 was (92.23 ± 4.5 and > 100), while pyocyanin from PsE02 was (85.47 ± 4.1 and > 100) against normal cells WI-38 and WISH, respectively. Pyocyanin from PsC05 exhibited moderate cytotoxic activity against the tested cell lines, particularly MCF-7 and HCT-116. It exhibited minimal cytotoxicity on the normal cells. Meanwhile, pyocyanin from PsE02 displayed weak activity towards all cells except for moderate activity towards MCF-7 (Table 2, Fig. S2).

Zhao and coauthors reported that pyocyanin caused 67% reduction in the number of HEPG2 cells compared to control [13]. They found that pyocyanin induced cell death via oxidative stress caused by an increase in (ROS), initiation of caspase-3, DNA damage, senescence acceleration and apoptosis. Another research indicated that pyocyanin exhibited reduced hemolytic activity at concentrations of 1 µg/ml. Furthermore, pyocyanin, at doses ranging from 6.25 to 100 µg/ml, was not cytotoxic to normal cell lines. Even at high concentration, there is still 80% or more cell viability, demonstrating that pyocyanin is safe for human ingestion in food [41]. Moreover, a previous study has indicated that pyocyanin treatment resulted in significant reduction and induced apoptosis/necrosis of human pancreatic cell line (Panc-1) in a concentration dependent manner. Consistent with our results, pyocyanin from clinical isolate was more active and more potent than soil isolate with wide safety margin [44].

## Conclusion

Our investigation has uncovered that *P. aeruginosa*, isolated from both clinical and environmental sources in Dakahlya governorate, Egypt, produces pyocyanin pigment in varying concentrations. Pyocyanin exhibits exceptional anti-bacterial, anti-fungal properties, and anti-biofilm activity; thereby enhancing its applicability medically and clinically. It could be used to prevent and/or eradicate biofilm from the surfaces of medical devices which is a chief source of nosocomial infections. Furthermore, its antioxidant along with its cytotoxic activity against cancer cell lines and minimal impact on normal cell lines, make it a promising contender for use as a substitute for synthetic agents in cancer treatment. However, additional toxicity tests of the extracted pyocyanin are necessary to establish a basis for its regulatory approval.

### Electronic supplementary material

Below is the link to the electronic supplementary material.

Supplementary Material 1. Fig S1. Pyocyanin yield among clinical isolates (A) and environmental isolates (B) of *P. aeruginosa*

Supplementary Material 2. Fig S1. Antitumor activity of purified pyocyanin from PsC05 (A) and PsE02 (B) isolates, and doxorubicin (C) against normal and carcinoma cell lines. IC_50_ values of purified pyocyanin from PsC05 and PsE02 isolates, and doxorubicin against normal and carcinoma cell lines (D)

Supplementary Material 3. Table (S1). Source and Pyocyanin yield of *P. aeruginosa* isolates in this study

Supplementary Material 4. Table (S2). Antimicrobial activity of purified pyocyanin from PsC05 and PsE02 isolates against food-borne pathogens, and human pathogenic microorganisms

Supplementary Material 5. Table (S3). Inhibition of biofilm formation by purified pyocyanin from PsC05 and PsE02 isolates against food-borne pathogens, and human pathogenic microorganisms

Supplementary Material 6. Table (S4). Eradication of Pre-formed Biofilm by purified pyocyanin from PsC05 and PsE02 isolates against food-borne pathogens, and human pathogenic microorganisms

## Data Availability

All data generated or analyzed during this study are included in this published article and its supplementary information files.

## Abbreviations

*P. aeruginosa*: *Pseudomonas aeruginosa*
ROS: reactive oxygen species
MHB: Mueller Hinton Broth
TLC: Thin Layer Chromatography
NMR: Nuclear Magnetic Resonance
FTIR: Fourier Transform Infra-Red Spectroscopy
MIC: minimum inhibitory concentration
TSBG: tryptic soy broth supplemented with 1% anhydrous glucose
FRAP: Ferric Reducing Antioxidant Potential
MTT: 3-(4,5-Dimethylthiazol-2- yl)-2,5-Diphenyltetrazolium Bromide
