# Supplementary figures and images for: Molecular and biological characterization of pyocyanin from clinical and environmental Pseudomonas aeruginosa
Source: Microb Cell Fact. 2023 Aug 29;22:166. doi: 10.1186/s12934-023-02169-0 (PMC10466709; doi:10.1186/s12934-023-02169-0)

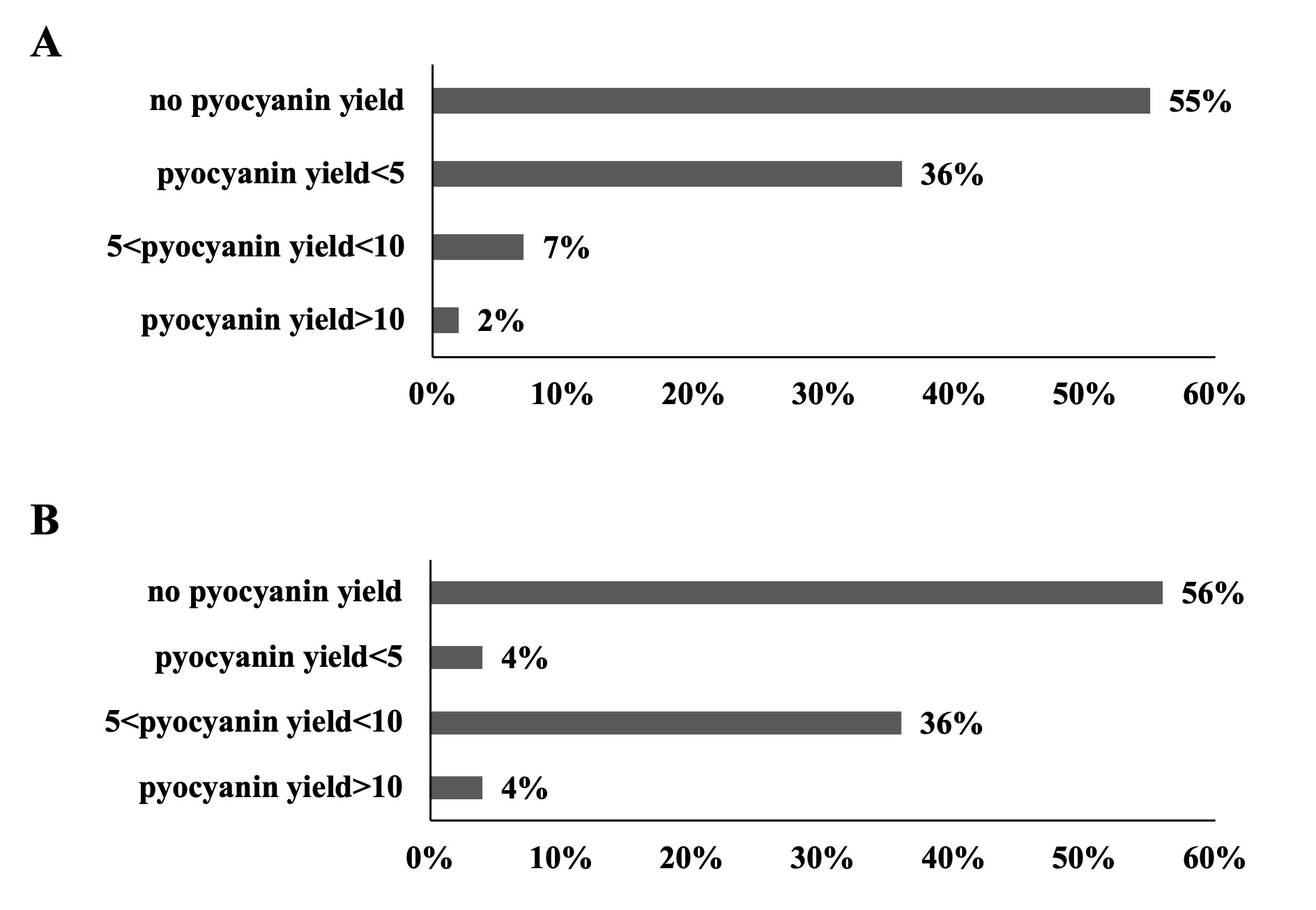

Supplement: Supplementary file 1 — Supplementary Material 1. Fig S1. Pyocyanin yield among clinical isolates (A) and environmental isolates (B) of P. aeruginosa [file 12934_2023_2169_MOESM1_ESM.jpg]

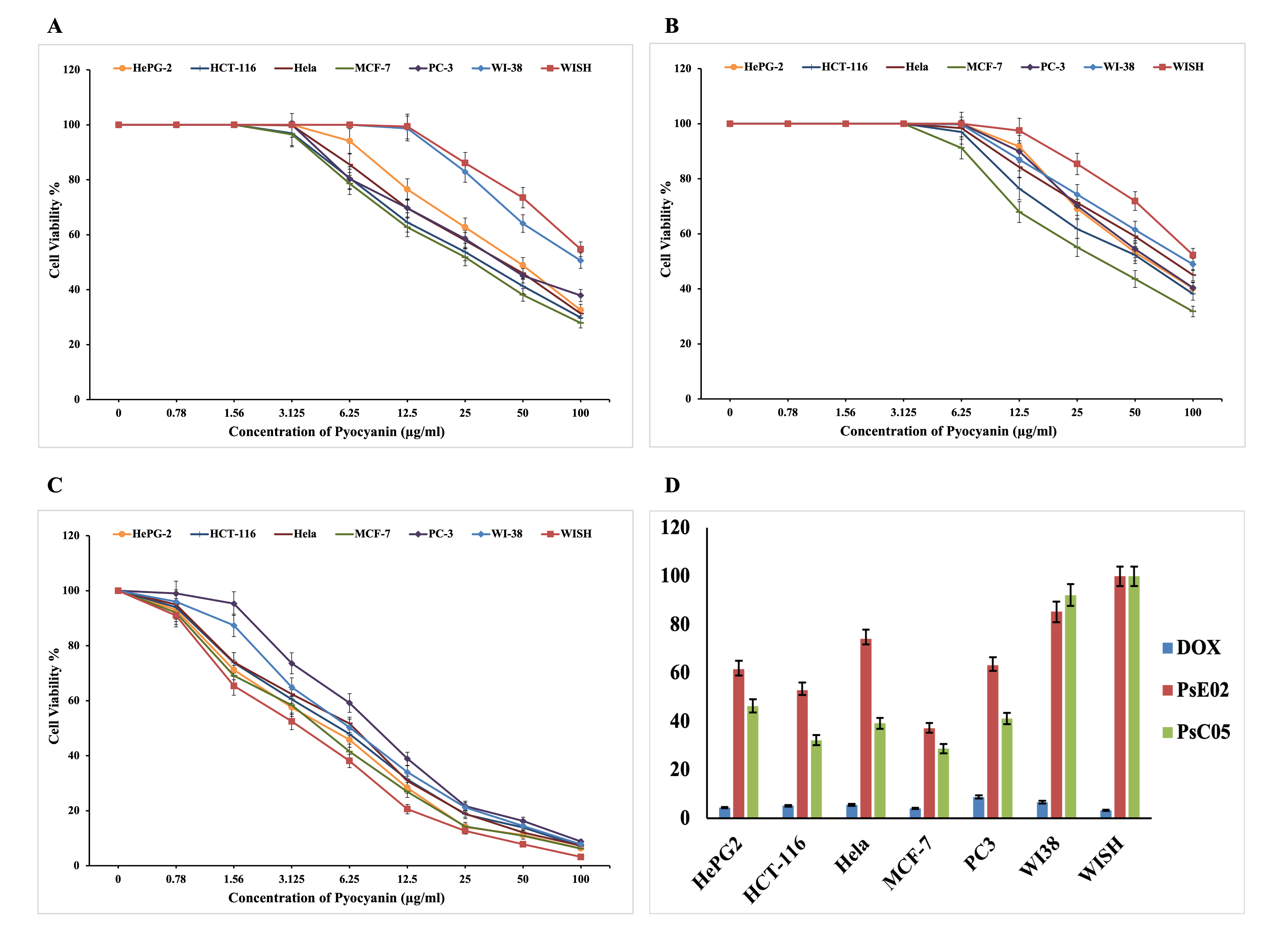

Supplement: Supplementary file 2 — Supplementary Material 2. Fig S1. Antitumor activity of purified pyocyanin from PsC05 (A) and PsE02 (B) isolates, and doxorubicin (C) against normal and carcinoma cell lines. IC50 values of purified pyocyanin from PsC05 and PsE02 isolates, and doxorubicin against normal and carcinoma cell lines (D) [file 12934_2023_2169_MOESM2_ESM.png]
