# Supplementary material for: Molecular and biological characterization of pyocyanin from clinical and environmental Pseudomonas aeruginosa
Source: Microb Cell Fact. 2023 Aug 29;22:166. doi: 10.1186/s12934-023-02169-0 (PMC10466709; doi:10.1186/s12934-023-02169-0)
Supplement: Supplementary file 3 — Supplementary Material 3. Table (S1). Source and Pyocyanin yield of P. aeruginosa isolates in this study [file 12934_2023_2169_MOESM3_ESM.docx]

**Table (S1):** **Source and Pyocyanin yield of *P. aeruginosa* isolates examined in this study.**

**A. Clinical isolates**

| **Sample No.** | **Source** | **Pyocyanin (Conc. µg/ml)** | **Sample No.** | **Source** | **Pyocyanin (Conc. µg/ml)** |
| --- | --- | --- | --- | --- | --- |
| PsC01 | Urine | 7.22 | PsC64 | Wound | 3.44 |
| PsC02 | Urine | 7.28 | PsC65 | Swab | 3.89 |
| PsC03 | Urine | 2.16 | PsC66 | Wound | NP |
| PsC04 | Urine | 5.32 | PsC67 | Wound | NP |
| PsC05 | Urine | 10.85 | PsC68 | Blood | 1.99 |
| PsC06 | Urine | NP | PsC69 | Wound | 6.72 |
| PsC07 | Urine | NP | PsC70 | Blood | 1.84 |
| PsC08 | Urine | NP | PsC71 | Diabetic Wound | NP |
| PsC09 | Urine | 2.2 | PsC72 | Urine | 4.09 |
| PsC10 | Urine | 2.5 | PsC73 | Blood | NP |
| PsC11 | Urine | NP | PsC74 | Wound | NP |
| PsC12 | Urine | 2.91 | PsC75 | Urine Catheter | NP |
| PsC13 | Urine | 4.57 | PsC76 | Urine Catheter | 4.67 |
| PsC14 | Urine | NP | PsC77 | Wound | NP |
| PsC15 | Urine | 4.83 | PsC78 | Wound | NP |
| PsC16 | Urine | NP | PsC79 | Blood | 6.67 |
| PsC17 | Urine | 2.52 | PsC80 | Wound | 2.78 |
| PsC18 | Urine | 4.16 | PsC81 | Wound | 1.8 |
| PsC19 | Urine | 2.45 | PsC82 | Wound | 2.93 |
| PsC20 | Urine | 4.89 | PsC83 | Swab | 3.34 |
| PsC21 | Urine | 6.04 | PsC84 | Wound | NP |
| PsC22 | Urine | 3.19 | PsC85 | Diabetic Wound | 3.02 |
| PsC23 | Urine | 5.82 | PsC86 | Wound | 2.13 |
| PsC24 | Urine | 2.61 | PsC87 | Urine Catheter | 3.17 |
| PsC25 | Urine | NP | PsC88 | Urine Catheter | NP |
| PsC26 | Urine | 5.51 | PsC89 | Urine | NP |
| PsC27 | Urine | 3.19 | PsC90 | Urine Catheter | 4.74 |
| PsC28 | Urine | NP | PsC91 | Wound | NP |
| PsC29 | Urine | 4.83 | PsC92 | Wound | 3.03 |
| PsC30 | Urine | NP | PsC93 | Blood | NP |
| PsC31 | Urine | NP | PsC94 | Wound | NP |
| PsC32 | Urine | 4.6 | PsC95 | Wound | NP |
| PsC33 | Urine | 10.4 | PsC96 | Wound | NP |
| PsC34 | Urine | NP | PsC97 | Blood | NP |
| PsC35 | Urine | 3.9 | PsC98 | Wound | NP |
| PsC36 | Urine | NP | PsC99 | Wound | NP |
| PsC37 | Urine | NP | PsC100 | Swab | NP |
| PsC38 | Urine | NP | PsC101 | Wound | NP |
| PsC39 | Urine | NP | PsC102 | Wound | NP |
| PsC40 | Urine | NP | PsC103 | Wound | NP |
| PsC41 | Urine | NP | PsC104 | Blood | NP |
| PsC42 | Urine | NP | PsC105 | Blood | 3.56 |
| PsC43 | Urine | NP | PsC106 | Blood | 2.88 |
| PsC44 | Urine | NP | PsC107 | Urine | NP |
| PsC45 | Urine | NP | PsC108 | Swab | 2.34 |
| PsC46 | Urine | 3.94 | PsC109 | Wound | 3.12 |
| PsC47 | Urine | 1.75 | PsC110 | Urine Catheter | NP |
| PsC48 | Urine | 3.89 | PsC111 | Wound | NP |
| PsC49 | Urine | 2.4 | PsC112 | Blood | NP |
| PsC50 | Urine | 1.7 | PsC113 | Wound | NP |
| PsC51 | Urine | 6.21 | PsC114 | Urine | NP |
| PsC52 | Urine | NP | PsC115 | Urine Catheter | NP |
| PsC53 | Urine | NP | PsC116 | Wound | 3.89 |
| PsC54 | Urine | NP | PsC117 | Wound | NP |
| PsC55 | Urine | NP | PsC118 | Wound | NP |
| PsC56 | Urine | NP | PsC119 | Blood | NP |
| PsC57 | Urine | 3.9 | PsC120 | Urine Catheter | NP |
| PsC58 | Urine | 4.81 | PsC121 | Urine | NP |
| PsC59 | Urine | NP | PsC122 | Urine Catheter | 4.66 |
| PsC60 | Urine | 2.68 | PsC123 | Wound | 3.56 |
| PsC61 | Urine | NP | PsC124 | Swab | NP |
| PsC62 | Urine | NP | PsC125 | Swab | NP |
| PsC63 | Wound | NP |  |  |  |

NP: Non-producer

**B. Environmental Isolates**

| **Sample No.** | **Source** | **Pyocyanin (Conc. µg/ml)** |
| --- | --- | --- |
| PsE01 | Soil | NP |
| PsE02 | Soil | 12.36 |
| PsE03 | Soil | NP |
| PsE04 | Soil | NP |
| PsE05 | Soil | NP |
| PsE06 | Soil | 8.62 |
| PsE07 | Soil | NP |
| PsE08 | Soil | 5.01 |
| PsE09 | Soil | NP |
| PsE10 | Soil | NP |
| PsE11 | Soil | NP |
| PsE12 | Soil | 5.18 |
| PsE13 | Soil | 9.21 |
| PsE14 | Soil | 7.8 |
| PsE15 | Soil | NP |
| PsE16 | Soil | 7.97 |
| PsE17 | Soil | 5.37 |
| PsE18 | Soil | 5.71 |
| PsE19 | Soil | 9.35 |
| PsE20 | Soil | NP |
| PsE21 | Soil | 4.33 |
| PsE22 | Soil | NP |
| PsE23 | Soil | NP |
| PsE24 | Soil | NP |
| PsE25 | Soil | NP |

NP: Non-producer
