# Supplementary material for: Molecular and biological characterization of pyocyanin from clinical and environmental Pseudomonas aeruginosa
Source: Microb Cell Fact. 2023 Aug 29;22:166. doi: 10.1186/s12934-023-02169-0 (PMC10466709; doi:10.1186/s12934-023-02169-0)
Supplement: Supplementary file 4 — Supplementary Material 4. Table (S2). Antimicrobial activity of purified pyocyanin from PsC05 and PsE02 isolates against food-borne pathogens, and human pathogenic microorganisms [file 12934_2023_2169_MOESM4_ESM.docx]

**Table (S2): Antimicrobial activity of purified pyocyanin from PsC05 and PsE02 isolates against food borne pathogens, and human pathogenic microorganisms.**

| **Source** | **Isolate** | **MIC of PsC05 pyocyanin (µg/ml)** | **MIC of PsE02 pyocyanin (µg/ml)** |
| --- | --- | --- | --- |
| **Foodborne pathogens** | *E. coli_1* | 40±0 | 20±0 |
|  | *E. coli_2* | 63.3±5.7 | 53.3±5.7 |
|  | *K. pneumoniae_1* | 183.3±5.7 | 123.3±5.7 |
|  | *K. pneumoniae_2* | 183.3±5.7 | 153.3±5.7 |
|  | *K. oxytoca_1* | 36.7±5.7 | 30±0 |
|  | *K. oxytoca_2* | 43.3±5.7 | 30±0 |
|  | *E. cloacae* | 63.3±5.7 | 53.3±5.7 |
|  | *E. aerogenes* | 183.3±5.7 | 153.3±5.7 |
| **Human pathogenic MDR/XDR Gram-Postive bacteria** | *S. aureus_*1_MDR | 116.7±5.7 | 103.3±5.7 |
|  | *S. aureus_*2_MDR | 96.7±5.7 | 123.3±5.7 |
|  | *S. pyogenes*_1_MDR | 140±10 | 123.3±5.7 |
|  | *S. pyogenes*_2_MDR | 116.7±5.7 | 103.3±5.7 |
|  | *S. agalactiae*_1_MDR | 116.7±5.7 | 123.3±5.7 |
|  | *S. agalactiae*_2_MDR | 150±10 | 140±10 |
|  | MRSA_1 | 66.7±5.7 | 46.7±5.7 |
|  | MRSA_2 | 50±0 | 40±0 |
|  | MRSA_3 | 66.7±5.7 | 50±0 |
| **Human pathogenic MDR/XDR Gram-Negative bacteria** | *E. coli_1_MDR* | 253.3±5.7 | 196.7±5.7 |
|  | *E. coli_2_MDR* | 196.7±5.7 | 146.7±5.7 |
|  | *K. pneumoniae_1_MDR* | 153.3±5.7 | 153.3±5.7 |
|  | *K. pneumoniae_2_MDR* | 146.7±5.7 | 196.7±5.7 |
|  | *P. mirabilis*_1_XDR | 300±10 | 203.3±5.7 |
|  | *P. mirabilis*_2_MDR | 250±10 | 196.7±5.7 |
|  | *A. baumannii*_1_MDR | 96.7±5.7 | 70±0 |
|  | *A. baumannii*_2_MDR | 103.3±5.7 | 103.3±5.7 |
|  | *A. baumannii*_3_MDR | 70±0 | 70±0 |
| **Human pathogenic *C. albicans*** | *C. albicans_1* | 253.3±5.7 | 246.7±5.7 |
|  | *C. albicans_2* | 296.7±5.7 | 296.7±5.7 |
|  | *C. albicans_3* | 303.3±5.7 | 246.7±5.7 |
|  | *C. albicans_4* | 296.7±5.7 | 296.7±5.7 |
