# Supplementary material for: Molecular and biological characterization of pyocyanin from clinical and environmental Pseudomonas aeruginosa
Source: Microb Cell Fact. 2023 Aug 29;22:166. doi: 10.1186/s12934-023-02169-0 (PMC10466709; doi:10.1186/s12934-023-02169-0)
Supplement: Supplementary file 5 — Supplementary Material 5. Table (S3). Inhibition of biofilm formation by purified pyocyanin from PsC05 and PsE02 isolates against food-borne pathogens, and human pathogenic microorganisms [file 12934_2023_2169_MOESM5_ESM.docx]

**Table (S3): Inhibition of biofilm formation by purified pyocyanin from PsC05 and PsE02 isolates against food borne pathogens, and human pathogenic microorganisms.**

|  |  | **% Reduction of formed biofilm by PsC05 pyocyanin** | | | | **% Reduction of formed biofilm by PsE02 pyocyanin** | | | |
| --- | --- | --- | --- | --- | --- | --- | --- | --- | --- |
| **Source** | **Isolate** | **½ MIC** | **¼ MIC** | **1/8 MIC** | **1/16 MIC** | **½ MIC** | **¼ MIC** | **1/8 MIC** | **1/16 MIC** |
| Foodborne pathogens | *E. coli_1* | 73.43 | 58.85 | 21.58 | 11.99 | 77.12 | 59.40 | 1.29 | 0.00 |
|  | *E. coli_2* | 61.09 | 55.06 | 12.05 | 0.82 | 64.10 | 52.05 | 20.54 | 0.00 |
|  | *K. pneumoniae_1* | 68.83 | 53.03 | 33.14 | 7.73 | 69.06 | 50.27 | 29.28 | 5.80 |
|  | *K. pneumoniae_2* | 72.65 | 56.83 | 21.87 | 10.46 | 72.07 | 55.07 | 6.83 | 4.29 |
|  | *K. oxytoca_1* | 63.70 | 62.52 | 6.79 | 3.74 | 60.42 | 44.02 | 14.05 | 5.15 |
|  | *K. oxytoca_2* | 66.41 | 39.05 | 11.19 | 2.98 | 58.20 | 45.77 | 31.59 | 0.00 |
| Human pathogenic MDR/XDR Gram-Postive bacteria | *S. aureus_*1_MDR | 64.84 | 43.88 | 2.80 | 2.60 | 62.44 | 45.63 | 14.84 | 0.00 |
|  | *S. aureus_*2_MDR | 66.90 | 40.40 | 16.16 | 15.16 | 68.01 | 44.44 | 12.79 | 9.09 |
|  | *S. pyogenes*_1_MDR | 76.45 | 57.39 | 11.43 | 10.08 | 74.88 | 52.01 | 12.33 | 9.64 |
|  | *S. pyogenes*_2_MDR | 69.30 | 52.17 | 27.36 | 2.30 | 63.42 | 48.84 | 16.62 | 7.92 |
|  | *S. agalactiae*_1_MDR | 66.80 | 56.14 | 21.51 | 7.17 | 68.85 | 35.45 | 25.00 | 24.38 |
|  | *S. agalactiae*_2_MDR | 66.52 | 49.15 | 31.77 | 3.81 | 69.91 | 47.88 | 31.99 | 0.00 |
| Human pathogenic MDR/XDR Gram-Negative bacteria | *E. coli_1_MDR* | 73.28 | 64.30 | 14.89 | 0.00 | 71.39 | 39.71 | 6.85 | 0.00 |
|  | *E. coli_2_MDR* | 80.45 | 52.87 | 17.62 | 15.70 | 70.68 | 58.46 | 7.15 | 2.26 |
|  | *K. pneumoniae_1_MDR* | 74.57 | 65.94 | 4.31 | 0.00 | 73.03 | 65.63 | 0.30 | 0.00 |
|  | *K. pneumoniae_2_MDR* | 81.92 | 68.96 | 17.47 | 5.70 | 83.23 | 69.67 | 12.36 | 0.00 |
|  | *P. mirabilis*_1_XDR | 69.48 | 66.14 | 0.00 | 0.00 | 49.22 | 40.31 | 0.00 | 0.00 |
|  | *P. mirabilis*_2_MDR | 74.32 | 67.26 | 11.75 | 10.66 | 60.57 | 45.75 | 0.00 | 0.00 |
|  | *A. baumannii*_1_MDR | 74.56 | 73.50 | 16.37 | 5.01 | 73.17 | 64.57 | 13.24 | 0.00 |
|  | *A. baumannii*_2_MDR | 65 | 47.95 | 6.14 | 3.07 | 67.39 | 53.59 | 13.50 | 6.10 |
|  | *A. baumannii*_3_MDR | 82.75 | 77.40 | 21 | 0.00 | 83.14 | 74.40 | 19.90 | 6.53 |
| Human pathogenic C. albicans | *C. albicans_1* | 33.92 | 24.73 | 0.00 | 0.00 | 38.52 | 11.30 | 0.00 | 0.00 |
|  | *C. albicans_2* | 49.58 | 23.28 | 4.90 | 0.40 | 54.79 | 35.61 | 5.75 | 0.00 |
