# Supplementary material for: Molecular and biological characterization of pyocyanin from clinical and environmental Pseudomonas aeruginosa
Source: Microb Cell Fact. 2023 Aug 29;22:166. doi: 10.1186/s12934-023-02169-0 (PMC10466709; doi:10.1186/s12934-023-02169-0)
Supplement: Supplementary file 6 — Supplementary Material 6. Table (S4). Eradication of Pre-formed Biofilm by purified pyocyanin from PsC05 and PsE02 isolates against food-borne pathogens, and human pathogenic microorganisms [file 12934_2023_2169_MOESM6_ESM.docx]

**Table (S4): Eradication of Preformed Biofilm by purified pyocyanin from PsC05 and PsE02 isolates against food borne pathogens, and human pathogenic microorganisms.**

|  |  | **% Reduction of preformed biofilm by PsC05 pyocyanin** | | | | **% Reduction of preformed biofilm by PsE02 pyocyanin** | | | |
| --- | --- | --- | --- | --- | --- | --- | --- | --- | --- |
| **Source** | **Isolate** | **½ MIC** | **¼ MIC** | **1/8 MIC** | **1/16 MIC** | **½ MIC** | **¼ MIC** | **1/8 MIC** | **1/16 MIC** |
| Foodborne pathogens | *E. coli_1* | 57.69 | 54.58 | 34.31 | 0.00 | 67.89 | 61.53 | 36.39 | 0.00 |
|  | *E. coli_2* | 58.82 | 34.11 | 6.35 | 0.00 | 61.88 | 32.00 | 17.88 | 14.11 |
|  | *K. pneumoniae_1* | 37.16 | 21.27 | 0.00 | 0.00 | 43.52 | 42.03 | 19.55 | 0.00 |
|  | *K. pneumoniae_2* | 61.30 | 46.49 | 24.52 | 0.00 | 74.36 | 58.75 | 19.10 | 0.00 |
|  | *K. oxytoca_1* | 52.32 | 41.56 | 13.93 | 0.00 | 61.12 | 46.21 | 3.18 | 1.88 |
|  | *K. oxytoca_2* | 62.35 | 53.84 | 27.65 | 18.98 | 57.93 | 40.09 | 32.56 | 3.10 |
| Human pathogenic MDR/XDR Gram-Postive bacteria | *S. aureus_*1_MDR | 72.65 | 59.63 | 0.00 | 0.00 | 76.44 | 58.15 | 0.00 | 0.00 |
|  | *S. aureus_*2_MDR | 59.79 | 42.55 | 5.48 | 1.56 | 58.48 | 30.28 | 0.00 | 0.00 |
|  | *S. pyogenes*_1_MDR | 66.66 | 53.71 | 20.76 | 0.00 | 68.95 | 45.71 | 12.95 | 1.33 |
|  | *S. pyogenes*_2_MDR | 50.90 | 41.42 | 9.81 | 0.00 | 60.72 | 55.55 | 8.52 | 0.00 |
|  | *S. agalactiae*_1_MDR | 70.35 | 56.14 | 15.84 | 9.30 | 75.54 | 68.71 | 10.92 | 0.00 |
|  | *S. agalactiae*_2_MDR | 57.61 | 48.03 | 14.44 | 4.08 | 67.97 | 55.25 | 16.32 | 0.00 |
| Human pathogenic MDR/XDR Gram-Negative bacteria | *E. coli_1_MDR* | 71.60 | 53.65 | 27.76 | 5.42 | 75.78 | 44.05 | 20.25 | 0.00 |
|  | *E. coli_2_MDR* | 71.92 | 59.96 | 28.24 | 8.32 | 64.64 | 58.92 | 33.62 | 6.75 |
|  | *K. pneumoniae_1_MDR* | 62.41 | 46.08 | 11.39 | 0.00 | 69.55 | 59.01 | 13.09 | 0.00 |
|  | *K. pneumoniae_2_MDR* | 67.65 | 55.84 | 15.26 | 8.53 | 74.64 | 64.16 | 20.18 | 0.00 |
|  | *P. mirabilis*_1_XDR | 52.60 | 51.56 | 0.00 | 0.00 | 62.97 | 55.72 | 0.00 | 0.00 |
|  | *P. mirabilis*_2_MDR | 51.71 | 48.66 | 0.00 | 0.00 | 57.80 | 55.61 | 0.00 | 0.00 |
|  | *A. baumannii*_1_MDR | 66.06 | 47.55 | 1.80 | 0.00 | 66.48 | 42.56 | 0.00 | 0.00 |
|  | *A. baumannii*_2_MDR | 63 | 51.94 | 5.46 | 0.00 | 60.28 | 56.54 | 7.19 | 0.00 |
|  | *A. baumannii*_3_MDR | 72.35 | 64.02 | 9 | 5.03 | 69.52 | 65.23 | 20.45 | 3.31 |
| Human pathogenic C. albicans | *C. albicans_1* | 28.15 | 24.92 | 0.00 | 0.00 | 25.21 | 12.90 | 0.00 | 0.00 |
|  | *C. albicans_2* | 34.22 | 23.79 | 0.00 | 0.00 | 32.86 | 24.57 | 0.00 | 0.00 |
